# Treatment of *Trypanosoma evansi-*Infected Mice With *Eucalyptus camaldulensis* Led to a Change in Brain Response and Spleen Immunomodulation

**DOI:** 10.3389/fmicb.2022.833520

**Published:** 2022-03-21

**Authors:** Mohamed A. Dkhil, Esam M. Al-Shaebi, Rewaida Abdel-Gaber, Abdulsalam Alkhudhayri, Felwa A. Thagfan, Saleh Al-Quraishy

**Affiliations:** ^1^Department of Zoology and Entomology, Faculty of Science, Helwan University, Cairo, Egypt; ^2^Department of Zoology, College of Science, King Saud University, Riyadh, Saudi Arabia; ^3^Department of Biology, College of Science, University of Hafr Al Batin, Hafr Al Batin, Saudi Arabia; ^4^Department of Biology, College of Science, Princess Nourah Bint Abdulrahman University, Riyadh, Saudi Arabia

**Keywords:** Surra, *Trypanosoma*, brain, spleen, *Eucalyptus camaldulensis*

## Abstract

Surra is a parasitic disease caused by the eukaryotic, unicellular hemoprotozoan, *Trypanosoma evansi*, which affects the development of animal production and is widespread among both domestic and wild animals. As such, in this research, we studied the antiparasitic activity and the ameliorative impact of *Eucalyptus camaldulensis* leaf extracts (ELE) against *T. evansi*-induced brain injury and spleen immune response in mice. As a result, we found that ELE decreased the amount of trypanosomes in the blood and improved the weight loss caused by infection. In addition, ELE reduced the parasite-induced brain and spleen histopathological damage. The parasite affected the levels of dopamine and serotonin, but after treatment with ELE, their concentrations significantly decreased to 154 ± 7 and 258 ± 11 μg/g, respectively. We clearly observed the antioxidant activity of ELE because of its ability to increase the induced change in the brain’s total antioxidant capacity and the nitric oxide level. The histopathological changes in the spleen also improved after ELE application. Based on our results, we concluded that ELE possesses antitrypanosomal antioxidant and protective effects in the brains of mice infected with *T. evansi*. Additional phytochemical screening and molecular studies are required to understand the mechanism underlying the effect of ELE.

## Introduction

Surra is a widespread hemoprotozoan disease caused by the infection of domestic and wild animals with the single-cellular eukaryote *Trypanosoma evansi* (Sazmand et al., 2022). Trypanosomosis poses a barrier to the development of animal production in many tropical regions of the world. The disease pattern generated by *T. evansi* is similar to that of *T. brucei gambiense*, which causes sleep sickness in humans. The pathogenicity of *T. evansi* varies across strains and animal species (De Menezes et al., 2004). The tsetse fly is considered the vector transmitting trypanosomes to the host, and trypanosomosis leads to the appearance of severe weakness and anemia, and the impairment of the nervous system (Desquesnes et al., 2013; Dkhil et al., 2021a). Human infection with *T. evansi* can also occur (Joshi et al., 2005; Powar et al., 2006). Symptoms of trypanosomosis can vary with animal species (Degneh et al., 2017). The increased parasite levels in the blood of the host disturb the level of neurotransmitters. Dkhil et al. (2021a) reported increased dopamine and serotonin levels in the brains of mice infected with *T. evansi*. In addition, epinephrine has been reported to be increased during *T. congolense* and *T. lewisi* infections (Sanchez, 1973; Kalu and Haruna, 1985).

Due to parasite resistance to currently used antitrypanosomal medications, scientists are looking for effective natural-resource-based therapies, such as natural products, to combat the infection.

*Eucalyptus camaldulensis* is a species of *Eucalyptus* that belongs to the Myrtaceae family and is known for its biologically active chemicals, such as alkaloids and flavonoids (Ghareeb et al., 2018). Extracts from this plant were used to treat animals suffering from malaria (Anigboro et al., 2020) and animal trypanosomosis (Kabiru et al., 2013), and it was reported to possess antioxidant properties (Shakibaie et al., 2021). *In vitro* studies carried out by Habila et al. (2010) showed that *E. camaldulensis* oil was effective against *T. evansi*.

To the best our knowledge, no reports have been published on the brain-protective effect and spleen-immune modulation of *E. camaldulensis* during *T. evansi* infection. As such, during trypanosomosis, we examined antioxidant activity in the brain as well as the status of neurotransmitters in mice. We also examined any morphological and histological alterations in the spleen.

## Materials and Methods

### Extract Preparation

We collected *E. camaldulensis* leaves from Qassim, Saudi Arabia. The samples were authenticated by a specialist from the herbarium of Helwan University. The leaves were cleaned, dried, and powdered. We macerated the obtained powder (100 g) by mixing it at 4°C for 24 h in 70% methanol. We filtered the obtained extract and then dried it using a vacuum evaporator (Lubbad et al., 2015). We used distilled water to dissolve the residue.

### Infrared Spectroscopy

For ELE analysis, we used a Nicolet 6700 Fourier-transform infrared spectroscopy (FT-IR) optical spectrometer from Thermo Scientific (Waltham, MA, United States). We mixed the powder of the extract (10 mg) with 100 mg of potassium bromide powder (1:99 wt%) to obtain a translucent sample disk that we then loaded into an FTIR spectroscope with a scan range of 400–4000 cm^–1^. The chemical bonds in a molecule can be determined by interpreting the infrared absorption spectra (Pakkirisamy et al., 2017).

### Flavonoid and Phenolic Content of *Eucalyptus camaldulensis* Leaf Extracts

Total flavonoid content was quantified using an aluminum chloride colorimetric assay, and total phenolic content was calculated using the Folin-Ciocalteu technique described by Lin and Tang (2007). The flavonoids in ELE were estimated using a calibration curve of quercetin, a standard flavonoid. A gallic acid standard curve was used to determine the phenolic concentration.

### Animals and Infection

We bred 25 C57Bl/6 female mice (11 ± 2 weeks old) under specific-pathogen-free conditions. They were housed in plastic cages under standard conditions of illumination with a 12-h light/dark cycle at 25 ± 1°C. Animals received a balanced diet and water *ad libitum*.

We maintained *T. evansi* in mice by weekly passage with infected blood. The mice were infected intraperitoneally with 10^3^ trypanosomes of *T. evansi* (Dkhil et al., 2019). We calculated the mean number of trypanosomes/5 fields.

The animals in the first group received only water by oral gavage, whereas the second group received ELE for 4 days (100 mg/kg). Three groups of animals (third to fifth) were infected and, 1 h later, the fourth group received 100 mg/kg ELE (Anigboro et al., 2020). The fifth group was treated with 1 mg/kg Cymelarsan (Merial, Lyon, France) (Hagos et al., 2010). We sampled the brains of the mice on day 4 post-infection after killing the animals by CO_2_ asphyxiation.

We dissected the brains of the mice, which were then washed twice in ice-cold 50 mM Tris-HCl to remove any blood. Subsequently, we carefully divided longitudinally each brain into two halves: we used the first half for histopathological analysis, and we weighed and immediately homogenized the second half in an ice-cold medium containing 50 mM Tris-HCl (pH 7.4) to yield a 10% (*w/v*) homogenate. We centrifuged the supernatants obtained from the homogenates at 1,000 × *g* for 10 min at 4°C to determine the concentrations of nitric oxide (NO), dopamine, and serotonin. We calculated body and spleen weights on day 4 p.i. We determined spleen index as described by Dkhil (2009) by calculating the ratio of spleen weight per milligram of mouse to the mouse weight per gram. The project was approved (approval No., HU2021/Z/AD/1121-01) by the Department of Zoology, Faculty of Science, Helwan University’s Committee of Research Ethics for Laboratory Animal Care.

### Histological Study

We fixed the brains and spleens of infected and non-infected mice in 10% formalin. After fixation, the specimens were dehydrated, embedded in wax, and sectioned into 5-μm-thick slices. Finally, we stained the sections with hematoxylin and eosin and then examined them. Spleen histology was semiquantified as described by Giamarellos-Bourboulis et al. (2006).

### Oxidative Status

According to Tsakiris et al. (2004), we prepared the brain homogenate to measure the total antioxidant capacity (TAC) using commercial kits (Biodiagnostic, Egypt) by the colorimetric method described in Koracevic et al. (2001). Additionally, we estimated the NO level according to Green et al. (1982).

### Dopamine and Serotonin

We weighed the brain and measured the levels of dopamine and serotonin as described by Ciarlone (1978).

### Statistical Analysis

We evaluated multiple variable comparisons using one-way ANOVA, and the data are presented as the mean standard error of the mean. We compared the significance between the classes using Duncan’s test. Statistical significance was set at *p* ≤ 0.05.

## Results

**FIGURE 1:**
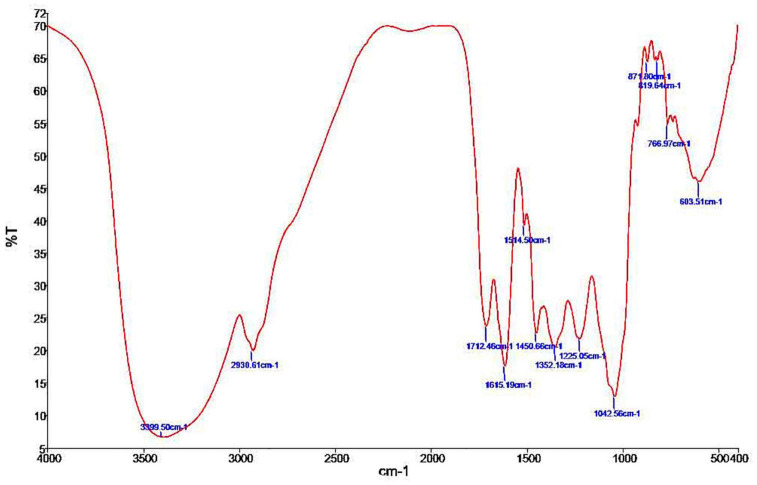
Phytochemical screening by FTIR spectroscopic analysis of *Eucalyptus camaldulensis* leaf extracts.

The FT-IR spectra for ELE are shown in Figure 1. We extracted Table 1 from the IR spectrum table.^1^ Our analysis of ELE using FTIR showed strong peaks at 1,712.46, 1,615.19, 1,514.50, 1,225.05, 1,042.56, 871.80, 766.97, and 603.51 cm^–1^. Medium peaks appeared at 3,399.50, 2,930.61, 1,450.66, and 819.64 cm^–1^. The expected classes of compounds were aliphatic primary amines, alkanes, carboxylic acids, α,β-unsaturated ketones, nitro compounds, sulfonamides, alkyl aryl ethers, anhydrides, and halo compounds (Table 1).

**TABLE 1:** FT-IR spectrum of *Eucalyptus camaldulensis* extract.

Absorption (cm^–1^)	Appearance	Transmittance (%)	Group	Compound class
3,399.50	Medium	6.6	N–H stretching	Aliphatic primary amine
2,930.61	Medium	19.9	C–H stretching	Alkane
1,712.46	Strong	23.8	C = O stretching	Carboxylic acid
1,615.19	Strong	17.5	C = C stretching	α,β-unsaturated ketone
1,514.50	Strong	39.3	N–O stretching	Nitro compound
1,450.66	Medium	22.7	C–H bending	Alkane
1,352.18	Strong	20.5	S = O stretching	Sulfonamide
1,225.05	Strong	21.8	C–O stretching	Alkyl aryl ether
1,042.56	Strong, broad	12.9	CO–O–CO stretching	Anhydride
871.80	Strong	64.6	C–H bending	1,3-disubstituted
819.64	Medium	64.8	C = C bending	Alkene
766.97	Strong	55.1	C–H bending	1,2-disubstituted
603.51	Strong	46.1	C–Br stretching	Halo compound

*Eucalyptus camaldulensis* leaf extracts has a total flavonoid and phenol content of 3.2 QE/g and 29.8 GAE/mg, respectively.

**FIGURE 2:**
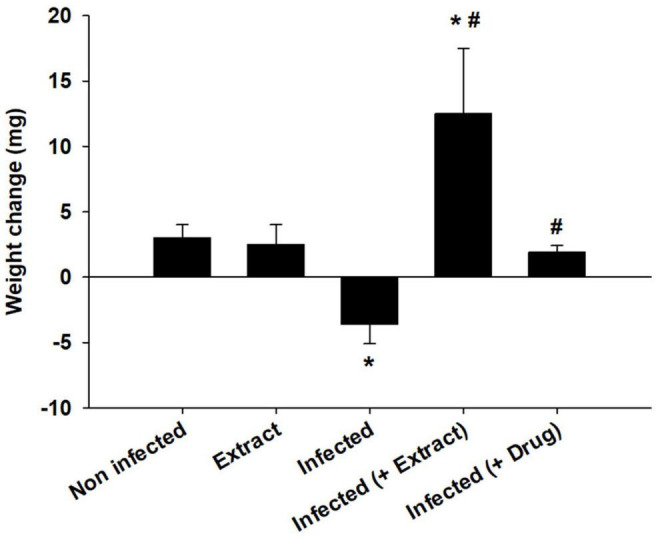
Effect of *E. camaldulensis* extract on body weight of mice infected with *T. evansi.* *p < 0.01, significance against the control group. #*p* < 0.01, significance against the infected group.

*Eucalyptus camaldulensis* leaf extracts (100 mg/kg) reduced the number of trypanosomes to approximately 57.2% (Table 2) on day 5 post-infection, whereas animals treated with 200 and 300 mg/kg ELE died on day 4 due to infection (Table 2). The parasite decreased the weight of the mice, but ELE increased their weight after treatment (Figure 2).

**TABLE 2:** Effect of *Eucalyptus camaldulensis* on the survival and trypanosomes number per 5 fields on day 5 post-infection.

Group	Survival	Trypanosomes/5 fields
Infected	Survived	645 ± 45
100	Survived	276 ± 70*
200	Dead	–
300	Dead	–
Drug	Survived	105 ± 10*

*Significance against the infected group at p ≤ 0.01.

**FIGURE 3:**
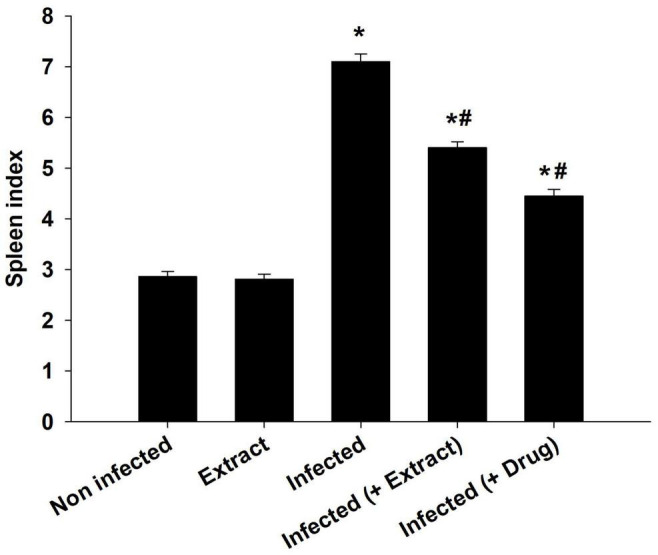
*E. camaldulensis* induced change in spleen index of mice infected with *T. evansi.* *p ≤ 0.01, significance against the control group. ^#^*p* ≤ 0.01, significance against the infected group.

**FIGURE 4:**
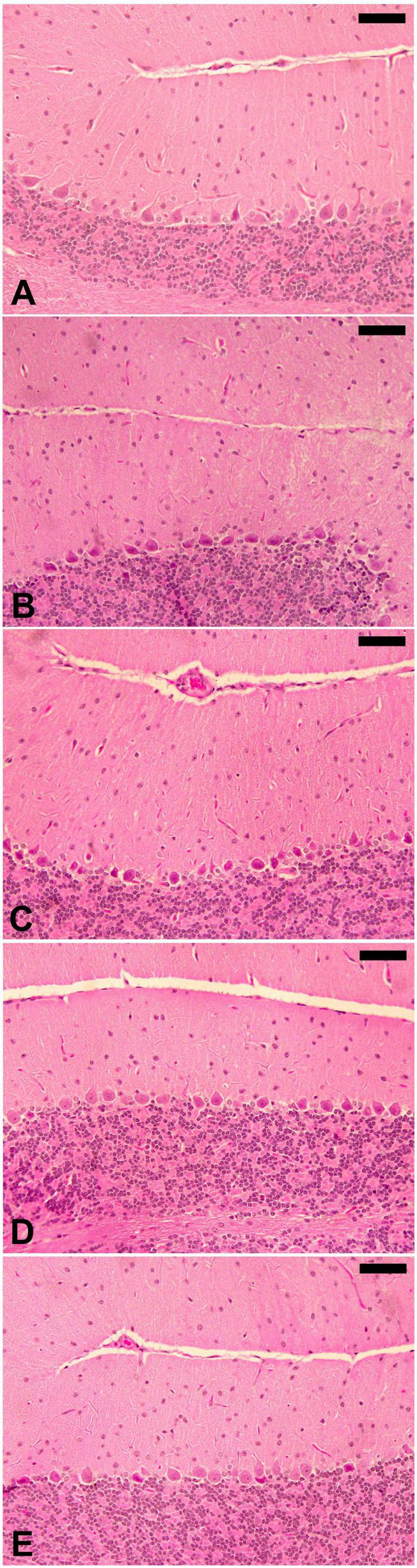
*E. camaldulensis* improves the brain histopathology after infection with *T. evansi*. Normal structure of control cerebellum **(A)** and ELE-treated **(B)** mice. **(C)** Infected cerebellum of mice with injured Purkinje cells and dilated sinusoids between the molecular layers. **(D)** ELE-treated infected cerebellum with improved structure. **(E)** Drug-treated cerebellum with improved structure. Scale bar = 25 μm.

**FIGURE 5:**
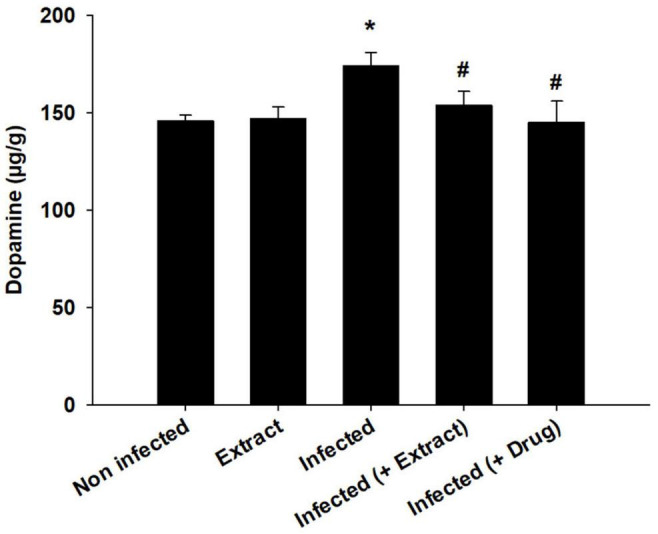
Change in brain dopamine of mice infected with *T. evansi* and treated with *E. camaldulensis*. *, significance against control group at *p* ≤ 0.01. ^#^, significance against infected group at *p* ≤ 0.01.

*Trypanosoma evansi* infection generated significant neurohistopathological alterations in Purkinje cells, including inflammation, bleeding, and structural abnormalities. In addition, infection reduced the number of cells in the Purkinje layer (Figure 3). Because of the parasite, dopamine and serotonin levels were much higher, but after treatment with ELE, the levels of these neurotransmitters significantly decreased (Figures 4, 5).

**FIGURE 6:**
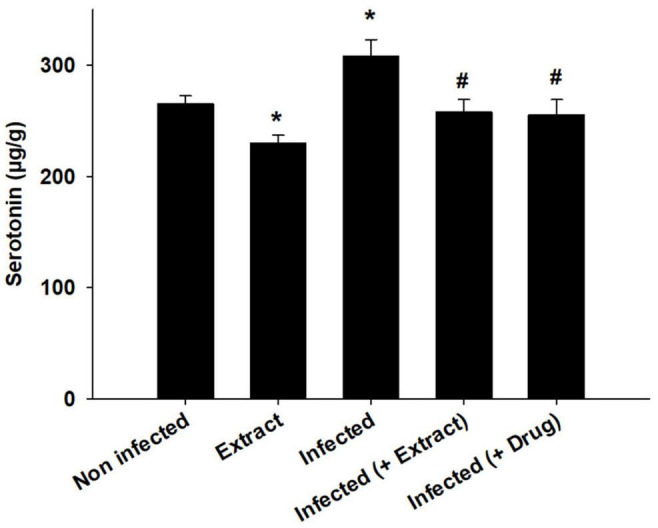
*E. camaldulensis* induced change in the level of Serotonin of mice infected with *T. evansi*. *, significance against control group at *p* ≤ 0.01. ^#^, significance against infected group at *p* ≤ 0.01.

**FIGURE 7:**
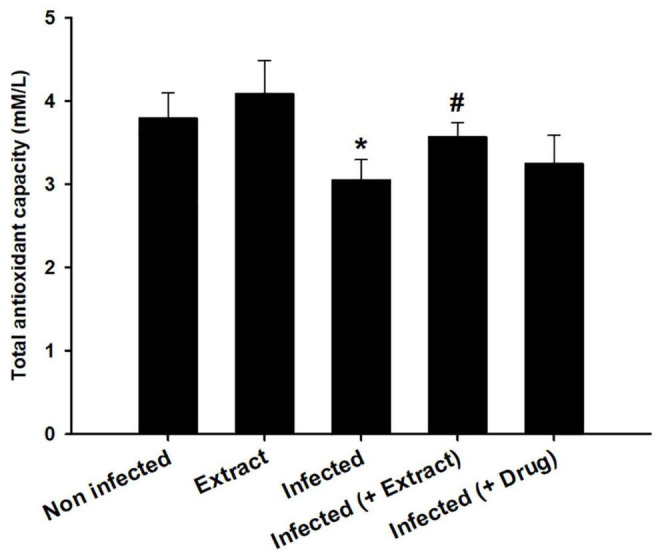
Effect of *E. camaldulensis* on brain total antioxidant capacity of mice infected with *T. evansi*. *, significance against control group at *p* ≤ 0.01. ^#^, significance against infected group at *p* ≤ 0.01.

We found that the infection decreased the TAC, but treatment with ELE ameliorated this decrease (Figure 6). Moreover, the level of NO decreased after treatment of the infected mice with ELE (Figure 7).

**FIGURE 8:**
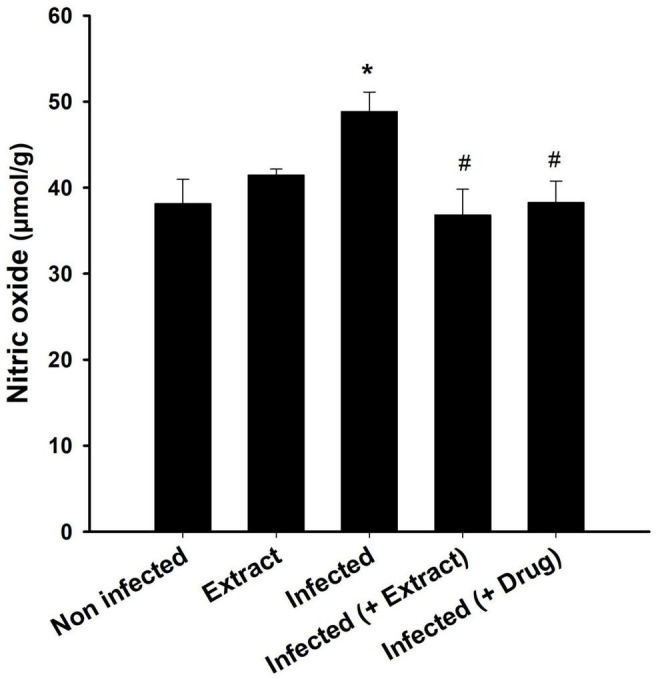
Brain nitric oxide level after treatment of *T. evansi*-infected mice with *E. camaldulensis*. *, significance against control group at *p* ≤ 0.01. ^#^, significance against infected group at *p* ≤ 0.01.

**FIGURE 9:**
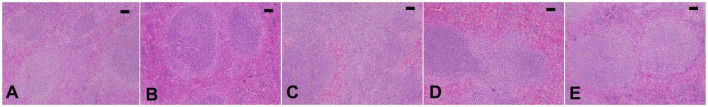
Spleen histology of mice. Normal structure of control spleen **(A)** and ELE-treated **(B)** mice. **(C)** Infected spleen of mice with dilated and fused white and red pulps and increased apoptotic bodies. **(D)** ELE-treated infected spleen with improved structure. **(E)** Drug-treated spleen with improved structure. Scale bar = 50 μm.

**FIGURE 10:**
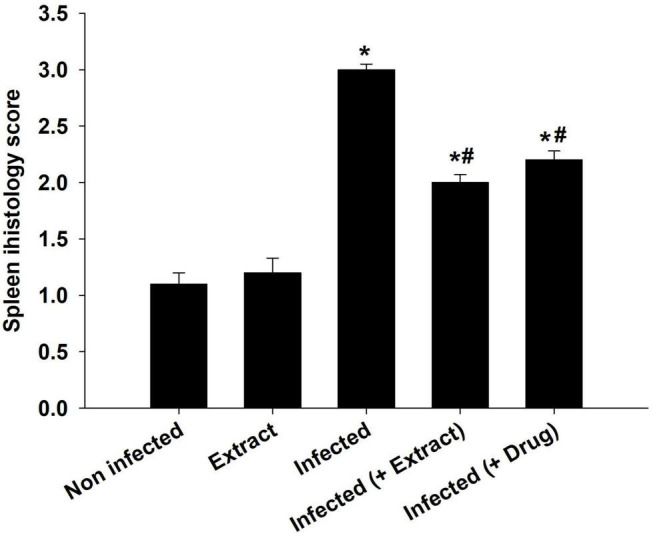
Spleen histology score of mice. *, significance against control group at *p* ≤ 0.01. ^#^, significance against infected group at *p* ≤ 0.01.

The infection caused marked changes in the spleen. Splenomegaly was pronounced when the spleen index increased approximately twofold (Figure 8). Our examination of spleen sections showed that the architecture of the spleen’s red and white pulps changed after infection (Figure 9). ELE treatment improved the induced histopathological changes. This was observed through the calculated spleen histology score, which indicated an ameliorative effect similar to that of the reference drug (Figure 10).

## Discussion

Trypanosomosis continues to be a serious health issue in both humans and animals, characterized by a complicated relationship between the host’s responses and the parasite’s actions, particularly the parasite’s generation of oxidative stress. This oxidative damage caused by parasite activity is considered to be the result of the physiological, histological, and biochemical changes that occur during infection.

*Eucalyptus camaldulensis* leaf extracts contains phenolic and flavonoid active compounds capable of suppressing the number of trypanosomes in mouse blood. Similar compound classes present in other plants, such as *Indigofera oblongifolia*, were found to have antitrypanosomal effects (Dkhil et al., 2019). Due to the presence of active phenolic compounds, *E. camaldulensis* shows significant antibacterial and antischistosomal activity (Ghareeb et al., 2018). The histopathological effects on the brain were induced by the rapid penetration of the trypanosome and its metabolites (Biswas et al., 2010), causing inflammation (Berlin et al., 2009). Few investigations on cerebral trypanosomosis have been conducted in Surra; however, in our prior work, we described the behavioral and histological effects of *T. evansi* in mice (Dkhil et al., 2021a). In addition to the histopathological effects on the brain, *T. evansi* causes liver and spleen injury (Biswas et al., 2010). In this study, splenomegaly with histopathological damage was pronounced, especially in the disorganization of white and red pulps.

Due to the enrichment of ELE with active compounds and the antioxidant properties of these compounds, both brain and spleen histology improved after treatment of *T. evansi*-infected mice.

In this study, we found that brain dopamine and serotonin levels increased after infection. The disturbance in dopamine and serotonin due to infection was also investigated by Amole et al. (1989) after infection with *T. brucei brucei*, which was attributed to the production of reactive oxygen species, which cause cellular damage and lead to the pathogenesis of the disease (Gupta et al., 2009).

Nitric oxide is measured for assessing immunological responses and oxidative stress levels (Chung et al., 2022). Activated macrophages create NO, which is harmful to a host of diseases, including *Trypanosoma* (Gazzinelli et al., 1992). Dkhil et al. (2021b) reported brain oxidative damage due to an increase in NO levels in mice infected with *T. evansi*. Increased NO production was reported in the brains of *T. brucei*-infected mice, suggesting that NO and its derivatives’ cytotoxicity may cause brain abnormalities (Keita et al., 2000). In our study, we found that ELE acted as an antioxidant agent that reduced oxidative damage in the brain (Nasr et al., 2019).

The spleen is the body’s largest secondary lymphoid organ, and it performs a variety of immunological processes in addition to its hematopoiesis and erythrocyte clearing functions. In this study, the increased spleen index and splenomegaly were evident during infection due to splenic cell hyperplasia and disease progression (Dkhil et al., 2019). Both spleen white and red pulps increase in size due to the response of macrophage activation, which occurs in the presence of the parasite. Furthermore, trypanosomes release toxins that disrupt organs and cause cell injury (Bal et al., 2012). This has an impact on the structure and function of the spleen. Similar improvements to those produced by ELE treatment after infection with *T. evansi* in the spleen in this study were reported in mice treated with *I. oblongifolia* extract (Dkhil et al., 2019).

The spleen is a key site for T-cell activation and B-cell differentiation. These activities are controlled in the brain (Whalley, 2020). Herr et al. (2017) reported that T-cell activation could be mediated by serotonin. In addition, Matt and Gaskill (2020) reported that dopamine plays an important role in immune function regulation. In this study, the neurotransmitters dopamine and serotonin increased after infection, leading to spleen cell activation. Because of the presence of phytoactive substances, ELE was able to regulate these alterations. We concluded that ELE has antitrypanosomal and immune-neuroprotective activities against *T. evansi* infection. However, further investigation into the molecular mechanisms underlying the neuroprotective and spleen response capabilities of *E. camaldulensis* is required.

## Data Availability Statement

The raw data supporting the conclusions of this article will be made available by the authors, without undue reservation.

## Ethics Statement

The animal study was reviewed and approved (Approval No., HU2021/Z/AD/1121-01) by the Department of Zoology, Faculty of Science, Helwan University’s Committee of Research Ethics for Laboratory Animal Care. Written informed consent was obtained from the owners for the participation of their animals in this study.

## Author Contributions

MD, EA-S, RA-G, and SA-Q designed the study. MD, EA-S, RA-G, and FT carried out the experiments and analyzed the data. All authors wrote and revised the manuscript.

## Conflict of Interest

The authors declare that the research was conducted in the absence of any commercial or financial relationships that could be construed as a potential conflict of interest.

## Publisher’s Note

All claims expressed in this article are solely those of the authors and do not necessarily represent those of their affiliated organizations, or those of the publisher, the editors and the reviewers. Any product that may be evaluated in this article, or claim that may be made by its manufacturer, is not guaranteed or endorsed by the publisher.

## Footnotes

1
sigmaaldrich.com
